# Optical Tweezers in Emulsion Research: Principles, Advances, and Prospects

**DOI:** 10.1021/acs.langmuir.5c05654

**Published:** 2026-02-05

**Authors:** Qifei Ma, Huaizhou Jin, Xiaoxiao Shang, Tamas Pardy, Ott Scheler, Simona Bartkova, Dan Cojoc, Denis Garoli, Shangzhong Jin

**Affiliations:** † College of Optical and Electronic Technology, 92270 China Jiliang University, Hangzhou 310018, China; ‡ Key Laboratory of Quantum Precision Measurement, College of Physics, 12377 Zhejiang University of Technology, Hangzhou 310058, China; § Thomas Johann Seebeck Department of Electronics, 54561 Tallinn University of Technology, Tallinn 12618, Estonia; ∥ Department of Chemistry and Biotechnology, Tallinn University of Technology, Tallinn 12618, Estonia; ⊥ CNR-Istituto Officina dei Materiali (CNR-IOM), SS 14 km 163.5, Area Science Park Basovizza, Trieste 34149, Italy; # 121451 Istituto Italiano di Tecnologia, Via Morego 30, Genova 16136, Italy; ¶ Università degli Studi di Modena e Reggio Emilia, Dipartimento di Scienze e Metodi dell’ingegneria, Via Amendola 2, Reggio Emilia 42122, Italy

## Abstract

Optical tweezers (OTs) have emerged as a powerful tool for probing emulsion dynamics with single-droplet precision, enabling quantitative analysis of interfacial interactions. Recent OT studies have systematically elucidated the critical factors governing emulsion stability, including ionic strength, pH, surfactant architecture, temperature, and photo/gas stimuli. Parallel advances in optofluidic control demonstrate that light-driven droplet rotation-achieved through angular momentum transfer and liquid crystal molecular reorientation represents a transformative approach for active soft matter manipulation. In this review, we conduct a systematic evaluation of OT systems, encompassing both instrumental configurations and cost-benefit analyses to assess their practical feasibility. The review critically examines the unique capabilities of OTs in emulsion research-including unprecedented spatial resolution and quantitative force measurement at the single-droplet level while addressing current limitations in throughput and operational complexity. Looking forward, the synergistic integration of OT technology with microfluidic platforms and machine learning algorithms is also presented.

## Graphical abstract

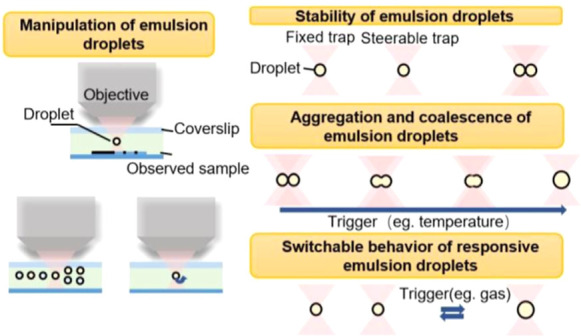

## Introduction

Emulsions are multiphase unstable systems consisting of two liquids that typically do not mix, along with an emulsifier that facilitates their mixing. One liquid forms the continuous phase, while the other is dispersed as small droplets. Essentially, there are three types of emulsions: oil dispersed in water (oil-in-water, O/W), water dispersed in oil (water-in-oil, W/O), and a more complex type known as multiple emulsions that can be prepared as water-in-oil-in-water (W/O/W) emulsions.^1,2^

Emulsions have diverse applications across industries, including food and beverage production,^3^ pharmaceutical formulations^4^ (e.g., drug delivery systems), and cosmetic products^5^ (e.g., creams, lotions, makeup). In biomedical research different emulsion systems are being applied as nanoscale (bio)­chemical reaction compartments for diagnostics^6^ and high-throughput screening of novel biomolecules.^7^ These applications highlight the importance of emulsions in various sectors, where they contribute to product quality, performance, and functionality.

Research aims to optimize emulsion properties for specific applications and explore novel formulations. Nowadays, techniques such as atomic force microscopy (AFM), magnetic tweezers (MTs), and OTs allow for the measurement of forces and displacements induced at the single-cell and single-molecule levels. These techniques have been employed to improve the experimental design for emulsion studies^8,9^ offering nanometer and submillisecond spatial and temporal resolution. The force is determined by measuring the displacement of a probe (such as a dielectric or magnetic microbead, or a cantilever tip), which is characterized by an elastic constant (stiffness). With stiffnesses typically in the range 10–10^5^ pN nm^–1^, AFM allows for the measurement of forces typically in the range 10–10^4^ pN. MT and OT probes typically have stiffnesses in the range 5 × 10^–3^–1 pN nm^–1^, making them suitable for measuring lower forces than AFM, typically in the range 0.1–200 pN. The manipulation, imaging, and measurement of forces by AFM exceeding 100 pN have primarily focused on droplets with diameters ranging from 20 to 200 μm, providing insights into the deformation process upon interaction with substrates or other droplets and surface tension.^10^ MT are commonly employed to measure low forces. However, in combination with microfluidics, microMT capable of generating μN forces have been developed to trap and extract magnetic particles from droplets, enabling physical separation in single cell-based droplets.^11^

Complementary to AFM and MT, OT allows for the manipulation and force measurement of dielectric particles without mechanical contact, making it the method of choice for studying many emulsion droplets with below 10 μm in diameter. OT enables the suspension of emulsion droplets in specified positions within the liquid, allowing for strict control over environmental conditions.^12–15^ Compared with the study by Huang et al.,^16^ this paper expands the discussion on OTs in emulsion research in an innovative and systematic way. It provides the first systematic evaluation and cost-benefit analysis for the configuration of OTs systems. While clarifying its unique advantages–such as high-resolution single-droplet imaging and quantitative force measurement–this review also acknowledges its inherent limitations in terms of throughput and operational complexity. Moreover, it offers a forward-looking summary of the future applications of OTs in emulsion research, proposing a new paradigm and a clear roadmap for advancing this field beyond traditional approaches.

Due to single droplet manipulation, low throughput requires extensive repetitions for statistical significance. Additionally, as variations in laser configurations (wavelength/power/NA) and calibration protocols across different laboratories, standardization challenges hinder data comparability. OTs present several key limitations in specific emulsion research.

(1) Manipulation of emulsion droplets: low torque efficiency and viscous resistance hinder stable droplet rotation, especially for nonspherical or heterogeneous systems.
(2) Stability mechanisms: difficulty in directly measuring microscopic forces (e.g., DLVO, steric) and capturing dynamic interfacial behaviors at high resolution.
(3) Coalescence studies: challenges in precisely triggering/merging thresholds and resolving fast (<ms) interfacial dynamics without laser-induced artifacts.
(4) Stimuli-responsive applications: poor synergy between optical stimuli and intrinsic emulsion responsiveness (e.g., pH/temperature), and limited scalability from single-droplet to bulk control.

OTs are expected to address key scientific questions, including studying nanoscale interfacial phenomena, manipulating active soft matter, emulating cellular processes and optimizing nanoemulsion-based drug delivery platforms, correlating microscopic interactions with macroscopic stability to enable rational emulsion design. Certainly, to address these scientific challenges, OTs systems should be enhanced through multimodal integration (combine with microfluidics,^17^ high-speed imaging and spectroscopy for real-time analysis^18^), advanced beam shaping (utilize holographic or Bessel beams for improved 3D manipulation and rotation control^19,20^ ).

In this review, we first introduce the principles of optical trapping and emulsion stability. Next, we discuss key applications in droplet manipulation, stability assessment, aggregation/coalescence dynamics, and the behavior of responsive emulsions, Figure 1 shows schematic summary of OTs applications in emulsion research. Subsequently, we examine OT instrumentation and its current limitations for emulsion studies. Finally, we conclude with an outlook on future challenges and opportunities.

**Figure 1:**
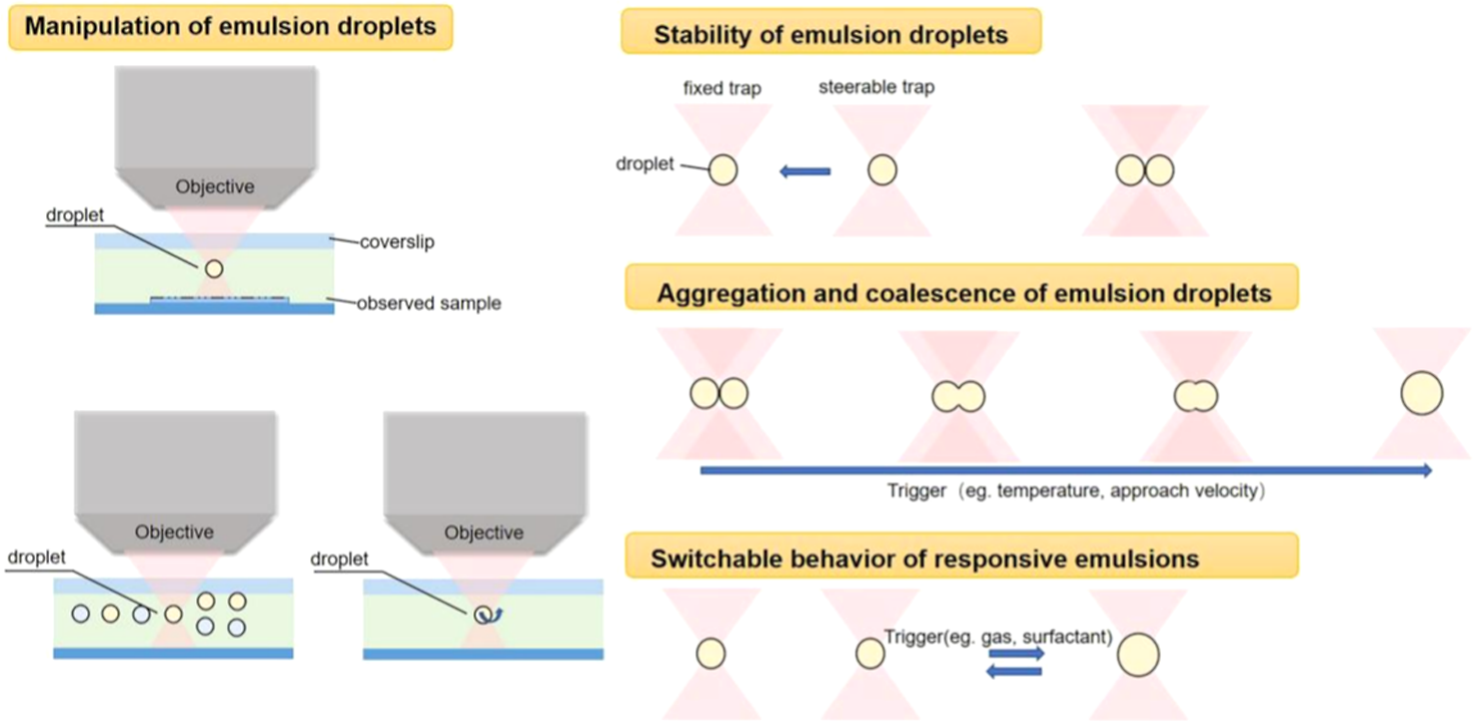
Graphical summary of OTs applications in emulsion research.

## Principle of Optical Trapping and Force Measurement by OT

The trapping and manipulation of dielectric microparticles using a single-beam gradient force optical trap was demonstrated by Ashkin and his group in 1986 ^21^ paving the way for new applications in physics, chemistry, nanobiotechnology, and biophysics. In recognition of his exceptional contribution, Ashkin was awarded the Nobel Prize in Physics 2018, “for the OTs and their application to biological systems”. The key to the single-beam gradient laser trap is the utilization of a microscope lens with a high numerical aperture (NA > 1). This lens allows the laser beam to be tightly focused, enabling the creation of a three-dimensional (3D) optical trap located near the focus of the lens, and thus extending the 2D trapping achieved by a single beam focused by a low NA lens (Figure 2A).

**Figure 2:**
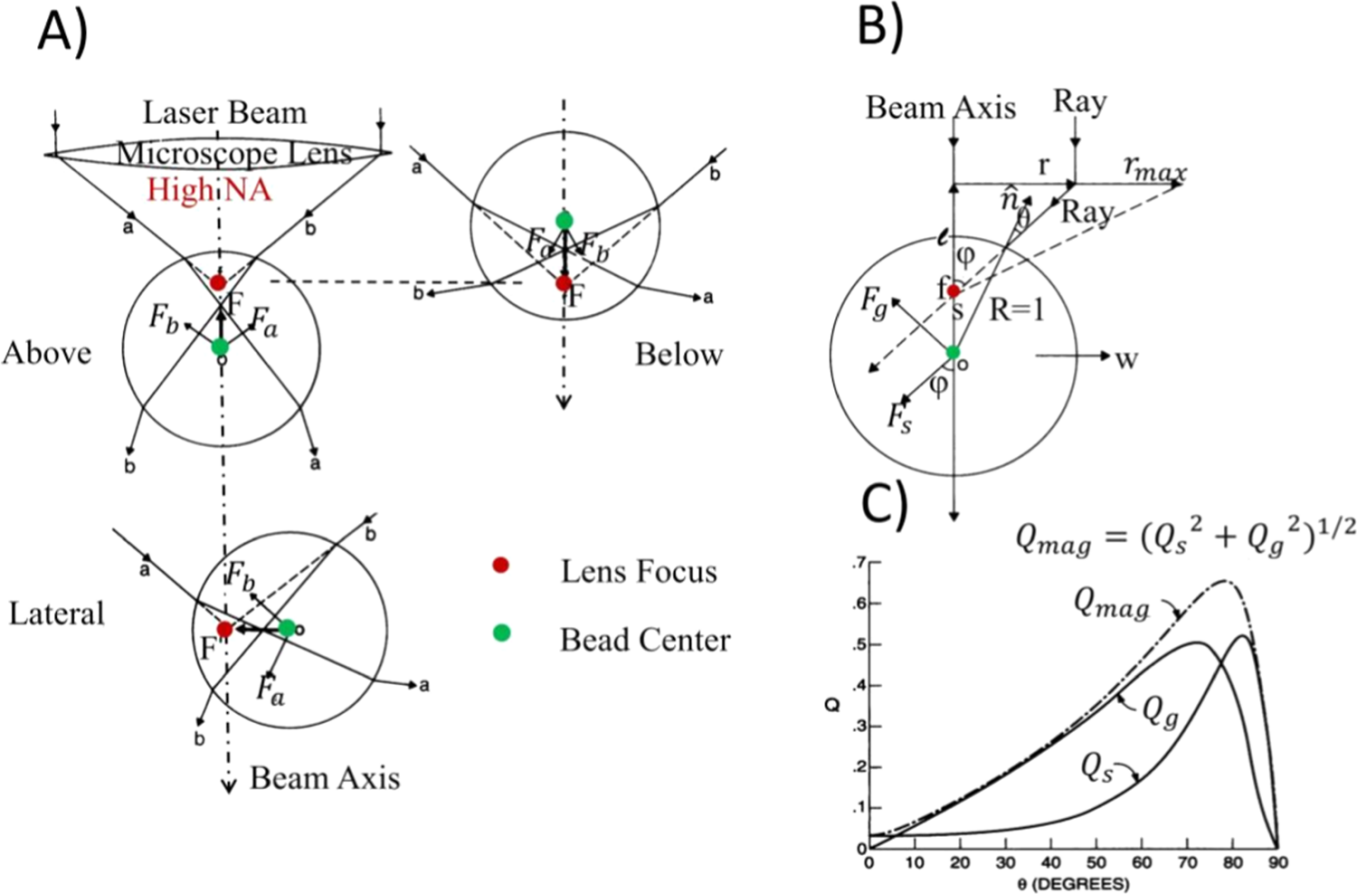
Illustrates the optical trapping model using ray optics. (A) Trapping of a dielectric microbead: the bead above the focus is pulled toward the lens focus; the bead below is pushed toward the focus, and the lateral bead is attracted toward the focus^24^ (B) Gradient and scattering force components arising from the refraction of an incident light ray.^24^ (C) Magnitude of the gradient and scattering force for a single ray, for a polystyrene bead in water, as a function the incidence angle. The magnitude of the resulting force Fmag–Qmag.^24^ The optical trapping and manipulation of dielectric particles of spherical shape and size larger than the wavelength of light can be readily explained using ray optics and the conservation of momentum of light (Adapted with permission from ref 24). Copyright 1992 Elsevier).

The Mie/ray-optics and Rayleigh/dipole approximations moment represent two limiting descriptions of the same trapping phenomenon.^22^ Ray optics provides an intuitive explanation of the trapping phenomenon for particles larger than the wavelength of light (2a > λ), emphasizing force balance and momentum flow. The dipole approximation applies for small particles in Rayleigh regime (a < λ), emphasizing effective potential and thermal fluctuations. Although both limits can be encompassed by full electromagnetic treatments,^23^ the dipole and ray-optics models remain indispensable for physical intuition and experimental design.

Using the ray optics approach, the light beam is decomposed into individual rays, each with its own intensity and polarization, propagating in straight lines through media with uniform refractive index. Each ray can change direction and polarization through reflection and refraction at dielectric interfaces, following Fresnel formulas. Considering a single ray of power P hitting the dielectric sphere at an angle of incidence θ with incident momentum per second of *n*_1_P/c (where *n*_1_ is the refractive index of the medium, and c is the speed of light), the force components^24^ are given by

1 $$F_{Z}=F_{S}=n_{1}P\left\{1+R\cos\;\cos2\theta -T^{2}\left[\cos\;\cos\left(2\theta -2r\right)+R\cos\;\cos2\theta \right]/\left(1+R^{2}+2R\cos\;\cos2r\right)\right\}/c$$

2 $$F_{Y}=F_{g}=n_{1}P\left\{R\sin\;\sin2\theta -T^{2}\left[\sin\;\sin\left(2\theta -2r\right)+R\sin\;\sin2\theta \right]/\left(1+R^{2}+2R\cos\;\cos2r\right)\right\}/c$$

where θ and *r* represent the angles of incidence and refraction, respectively, while *R* and *T* denote the Fresnel reflection and transmission coefficients (Figure 2B). The force component *F*_*z*_, pointing in the direction of the incident ray is called the scattering force *F*_s_ for a single ray, while the *F*_*y*_ component pointing in *a* direction perpendicular to the ray is denoted as the gradient force *F*_g_. For a laser beam, the scattering and gradient forces are defined by the vectorial sums of the scattering and gradient force contributions of the individual rays comprising the beam. The magnitude of the optical force is

$$F=Qn_{1}P/c$$

where

3 $$Q=\sqrt{Q_{S}^{2}+Q_{g}^{2}}$$

The dimensionless coefficient *Q* takes into account the material and shape of the particle and can have a value of maximum *Q* = 2.

For dielectric particles whose characteristic size a is much smaller than the optical wavelength λ, optical trapping can be described within the Rayleigh (dipole) approximation. In this regime, the optical field is approximately uniform across the particle, and the interaction with light is governed by an induced electric dipole moment, following the physical picture introduced by Ashkin for single-beam gradient-force trap.^22^ A tightly focused laser beam induces an electric dipole moment p in the particle, proportional to the local electric field E

4 p=αE

here α­(ω) denotes the particle polarizability, which is in general complex at optical frequencies. For weakly absorbing soft-matter particles, the trapping behavior is dominated by the real part of α. In the quasi-static Rayleigh limit, the real part of the polarizability of a small dielectric sphere is

5 $$Re\left(\alpha \right)=4\pi \varepsilon_{m}a^{3}\left(\varepsilon_{p}-\varepsilon_{m}\right)/\left(\varepsilon_{p}+2\varepsilon_{m}\right)$$

Particle’s permittivity is ε_*p*_, medium of permittivity is ε_*m*_.

The interaction of the induced dipole with the spatially varying optical field gives rise to a conservative gradient force. The cycle-averaged interaction energy is

6 $$U\left(r\right)=-\left(1/2\right)Re\left(\alpha \right)|E\left(r\right){|}^{2}$$

and the corresponding force is obtained as

7 $$F_{g}\left(r\right)=\left(1/2\right)Re\left(\alpha \right)\nabla |E\left(r\right){|}^{2}$$

For Re­(α) > 0, which is typical when the particle refractive index exceeds that of the surrounding medium, this force pulls the particle toward regions of higher intensity, i.e. toward the focal region of the beam.

The same induced dipole also radiates electromagnetic energy. The associated momentum transfer produces a scattering (radiation-pressure) force directed along the local propagation direction of the beam. This force is nonconservative and is responsible for pushing the particle downstream.

The relative importance of the gradient and scattering forces in the Rayleigh regime can be understood through a simple scaling argument, following Ashkin’s original reasoning. In the Rayleigh limit, the induced dipole moment scales with the particle volume, *p*∝αE with α∝*a* ^3^. The gradient force is proportional to the induced polarization and the intensity gradient, hence *F*_g_∝α∇*I*–*a* ^3^. By contrast, the scattering force is associated with the power reradiated by the induced dipole; since the scattered power scales as *p* ^2^, it follows that the scattering force scales as *F*_s_∝*p* ^2^∼α^2^∼*a* ^6^(for fixed wavelength and intensity). Therefore, as particle size decreases, the scattering contribution falls off much more rapidly than the gradient contribution, which is why stable single-beam trapping is particularly favorable in this regime.

In this dipole-based description, both trapping and radiation pressure arise from the same physical origin: the induced polarization of the particle by the optical field. The in-phase component of the response (Re­(α)) gives rise to a conservative gradient force and optical confinement, while the out-of-phase component leads to radiation and a scattering force. For larger particles, where the Rayleigh condition no longer holds, the dipole picture must be replaced by Mie or ray-optics descriptions, which embody the same physical principle of momentum transfer from light to matter. OTs operate by engineering the optical field so that the restoring gradient force dominates the scattering force near the focus, consistent with Ashkin’s original formulation.

Due to thermal motion, the position of the optically trapped particle fluctuates around the point of equilibrium, where the light intensity is maximum.^21^ For small distances from this point (typically 0–400 nm), the trap potential can be described as a harmonic/parabolic potential (Figure 3A), in which the trapped particle tends to reach the potential minimum. For a parabolic potential, the restoring force exerted on the particle is proportional to the position x, by an elastic constant k, called trap stiffness (Figure 3B), the optical trap behaving as a Hookean spring

8 $$F=-k_{trap}x$$

**Figure 3:**
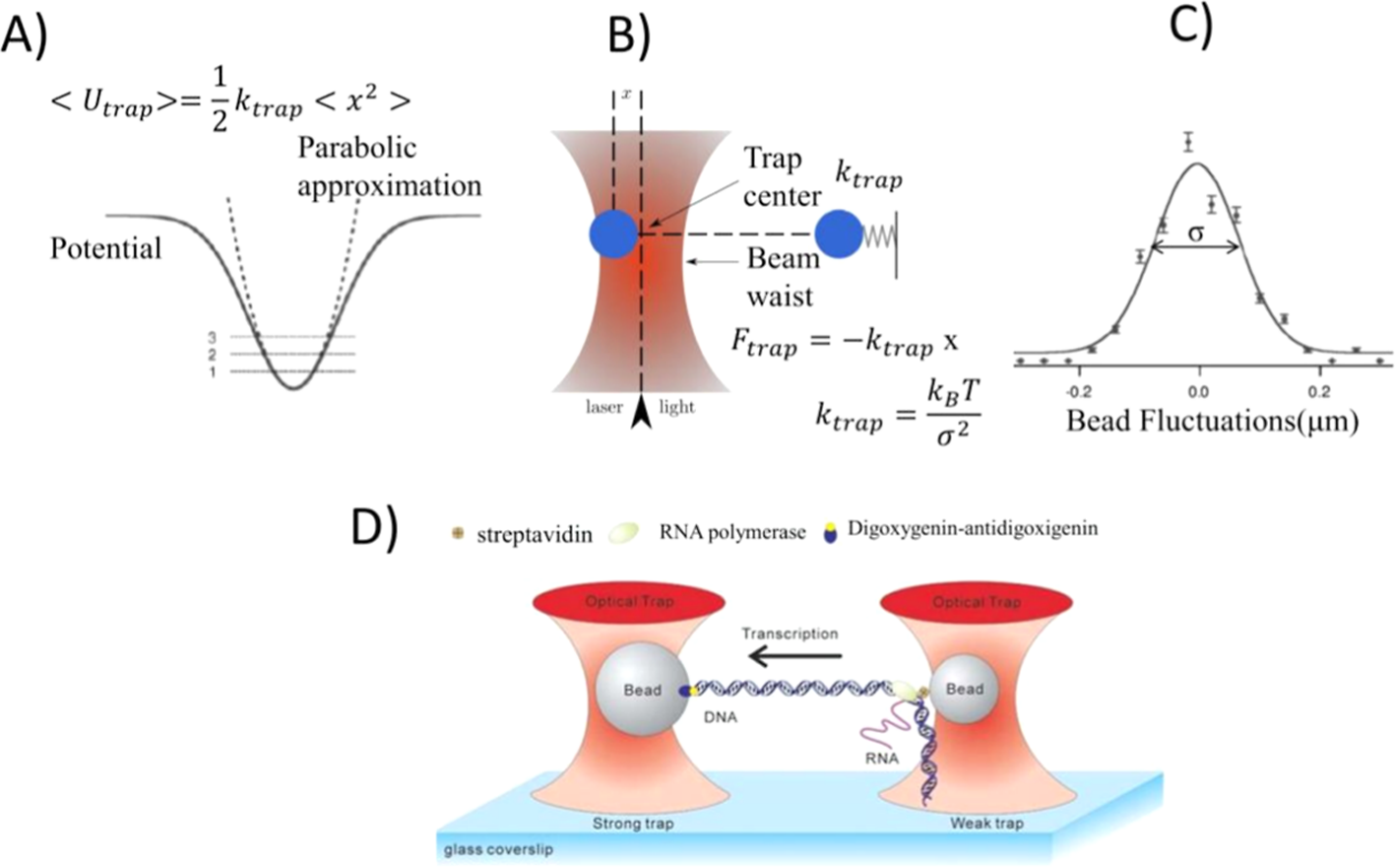
(A) Optical trap potential. (B) Optical trap as a Hookean spring. (C) Distribution of the bead position. (D) And example of a dual beam optical trap for DNA transcription experiment (Reproduced with permission from ref 120 Copyright 2006 Royal Society of Chemistry).

The value of the trap stiffness can be determined by tracking the position of the bead in the trap for 3–5 s at a frequency of about 5 kHz and using the Boltzmann distribution (Figure 3C) or the Equipartition theorem

9 $$k_{trap}=k_{B}T/\sigma^{2}$$

where σ^2^ = < *x* ^2^> is the variance, *k*_*B*_ the Boltzmann constant and *T* the temperature.

Aside of this method, there are also other techniques to calibrate the trap: passive and active power spectrum, drag force, light momentum change.^25,26^

A useful device for the study of emulsion droplets is the dual-beam OTs. This system employs two optical traps, allowing the trapping and independent manipulation of two different particles in liquid.^27^ While the primary biological application of dual-beam OT is measuring the biophysical properties of molecular motors, it can also be applied to droplets. In this setup, two trapped droplets can be brought together to interact, and the displacement of each droplet is measured separately. One advantage of this configuration is its higher spatial resolution compared to a single trap, which can be achieved even with droplets of larger diameters (>1 μm).

## Theory of Emulsion Stability

The interactions at micro scales of chemical reagents, solid particles, bubbles, droplets, and solid surfaces in complex fluids play a crucial role, which impact the macroscopic performance and efficiency of related engineering processes. Classical intermolecular and surface interactions include Derjaguin–Landau–Verwey–Overbeek (DLVO) interactions (i.e., van der Waals (VDW) and electric double layer (EDL) interactions) and non-DLVO interactions such as steric and hydrophobic interactions.^28^

### DLVO Theory

The DLVO theory^29^ has been widely applied to describe the stability of colloidal spheres in aqueous solution, which includes both VDW and EDL interactions.

VDW forces are ubiquitous between all molecules and surfaces, because VDW interactions result from the associated fluctuating electric dipole moments when two molecules approach each other. The VDW force^30^ can be calculated using eq 10.

10 $$F_{VDW}\left(h\right)=-A_{H}R/6h^{2}$$

where *A*_*H*_ is the Hamaker constant between two spheres (with radius *R*) in an aqueous solution, and *h* is the surface-to surface distance between two spheres.

An EDL is generated when a solution containing ions is in contact with a charged surface. In case of spherical particles, if spheres carry the same charge, the EDL surface interactions are repulsive, preventing the aggregation and precipitation of the spheres. It is more convenient to calculate the EDL force^31,32^ with the surface distance of spheres directly, which can be demonstrated using eq 11

11 $$F_{EDL}\left(h\right)=\left(e^{2}Z^{2}/8\pi \varepsilon_{0}\varepsilon_{r}R^{3}\right)\kappa^{-1}\exp\left(-\kappa h\right)$$

where *Z* is a process variable, *R* is the radius of spheres, ε_0_ is the permittivity of vacuum, ε_*r*_ is the relative permittivity of the aqueous phase solution, *e* is the elementary charge, and κ^–1^ is the Debye length. The Debye length can be calculated by eq 12

12 $$\kappa^{-1}=\sqrt{\left(\varepsilon_{0}\varepsilon_{r}k_{B}T\right)/\left(2\times 10^{3}NAe^{2}I\right)}$$

where *k*_*B*_ is the Boltzmann constant, *T* is the thermodynamic temperature, and *I* is the intensity of ions in aqueous solution. *I* can be calculated by eq 13

13 $$I=\frac{1}{2}\sum_{i}c_{i}{z_{i}}^{2}$$

where *c*_*i*_ is the concentration of ions, *z*_*i*_ is the electric charge of ions. *Z* can be calculated by eqs 14 and 15

14 $$Z=e\xi R/\left(k_{B}T\lambda_{b}\right)\left(1+\kappa R\right)$$

15 $$\lambda_{b}=e^{2}/\left(4\pi \varepsilon_{0}\varepsilon_{r}k_{B}T\right)$$

where ξ is the ξ-potential of spheres and λ_*b*_ is the Bjerrum length.

Hence, the total interaction force between two spheres can be calculated using eq 16.

16 $${F_{tol}\left(h\right)=F}_{VDW}\left(h\right)+F_{EDL}\left(h\right)$$

### Non-DLVO Theory

Besides DLVO interactions, there are several other interactions, referred as non-DLVO interactions, including steric force, depletion force, polymer bridging interaction, hydrophobic effects, and hydration force, which can also impact the interactions of particles.

- Steric force^33^ arises from the compression of polymer chains when two particles stabilized by polymers come into close proximity.
- Depletion force. For spheres stabilized by polymers, if the polymer that does not adsorb or weakly adsorb onto any surfaces of spheres during the approach of two spheres, the polymer between the spheres is squeezed out, leaving a bare surface, thus a “depletion zone” will appear. There is a difference in polymer concentration between the depletion zone and polymer solution, resulting in a difference in osmotic pressure, which causes water molecules to migrate from the depletion zone into the bulk solution, creating a depletion force.^34,35^

- Bridging force. For spheres stabilized by polymers, if relatively low amounts of polymers adsorb onto the surface of one sphere, the other ends of these polymers may bind to other spheres, resulting in adhesive bridging force^36,37^ between the two surfaces.
- Hydrophobic interactions. Hydrophobic molecules are nonpolar molecules, typically possessing long carbon chains that cause them to self-associate in aqueous solutions. Hydrophobic interactions^38^ can drive the aggregation of hydrophobic moieties in water mediums and adjust molecular or biomolecular conformation on a macro scale, as well as facilitate oil–water separation, which usually exist between different proteins and other biochemical molecules. For example, when proteins fold in water, they tend to bury hydrophobic groups and expose hydrophilic groups.

### Applications

In this section, applications of OTs with relation to emulsions will be comprehensively reviewed. OTs, a powerful tool in the field of biophysics and nanotechnology, have found diverse applications in manipulating and studying colloidal systems such as emulsions. To gauge the interest of OTs in various subfields, we present an overview of the number of publications indexed in Scopus, highlighting the growing interest and research contributions in this evolving area of study, as shown in Figure 4.

**Figure 4:**
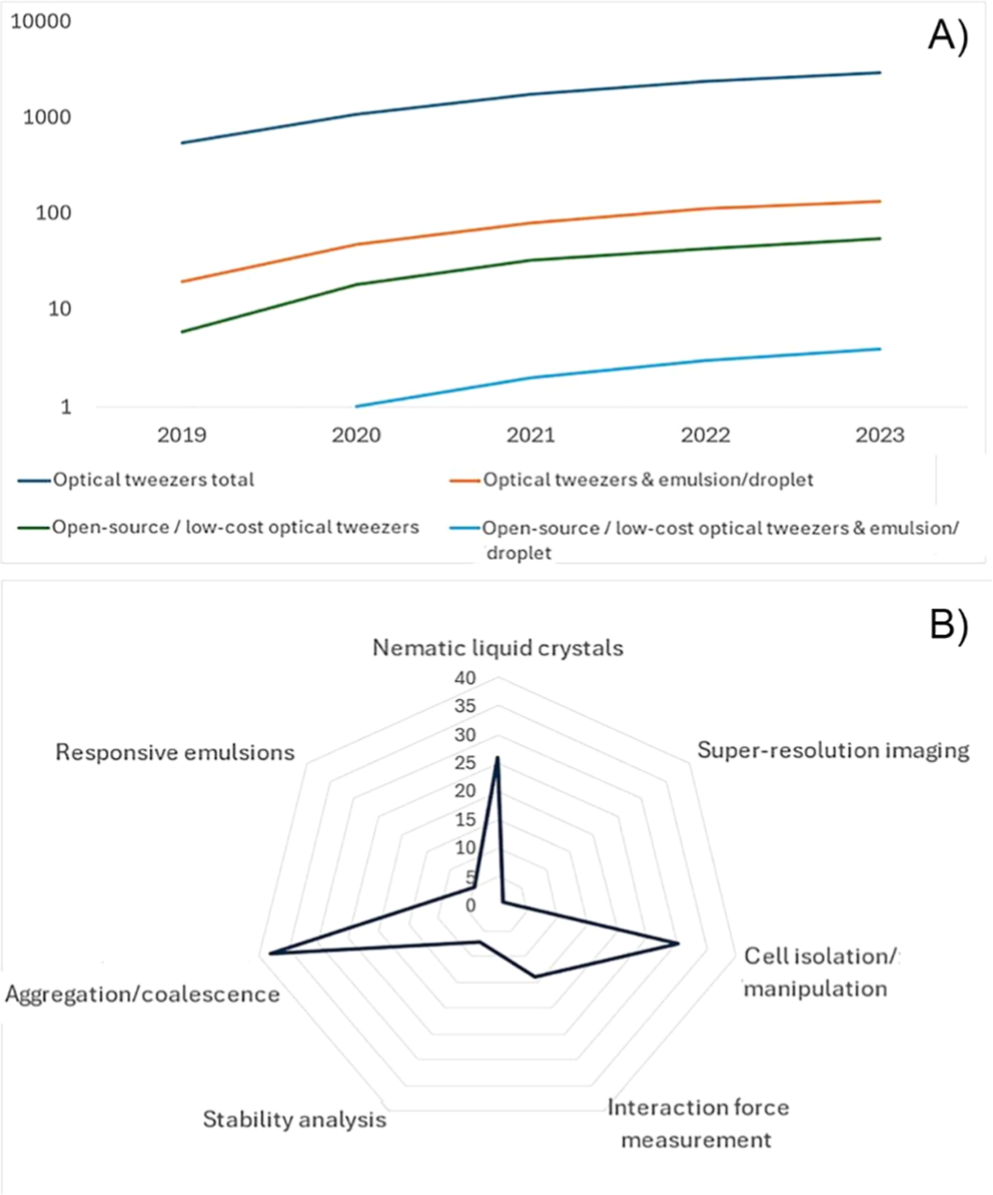
According to the Scopus publication index, there has been (A) a steadily rising interest in OTs in general, and a rapidly rising interest in emulsions/droplets within the last 5 years. Certain application areas (B), such as droplet aggregation/coalescence, NLCs and cell isolation/manipulation (in/with droplets) stand out in particular and will be discussed further in this review.

### Manipulation of Emulsion Droplets

The manipulation of droplets with OTs enables a range of applications, including: rotating droplets; leveraging their unique properties for high-resolution microscopic imaging; and integrating with Raman spectroscopy or microfluidics for droplet sorting and detection. Table 1 compared the application of manipulation of emulsion droplets.

**Table 1:** Comparison of Manipulation of Emulsion Droplets

droplet manipulation			droplet sorting and detection				microdroplet-assisted imaging			application
elliptically polarized laser light with OTs	metalens and OT	polarization-controlled holographic OTs	OTs and Raman spectroscopy	OTs and Raman spectroscopy	OTs and fluorescence imaging	OTs and microfluidic chip	OTs and microfluidic chip	OT and lipid droplets	OT and microsphere	OT configurations
NLC/cholesteric LC	NLC	birefringent cubic calcite microparticles	droplet	biological particles	10 μm fluorescent particles	droplet	droplets of any size	lipid droplets	microsphere	emulsion types
^52^	^51^	^50^	^48^	^47^	^46^	^45^	^43^	^42^	^40,41^	refs

Taking advantage of the ability of OTs to capture droplets, this technique, when combined with droplets, also finds important applications in imaging. To overcome the diffraction limit and magnify nanostructures, microsphere-assisted imaging^39^ has become an irreplaceable tool in life sciences and precision measurement due to advantages such as low cost and label-free operation. Wen et al.^40^ and Lin et al.^41^ combined OTs with microspheres, overcoming limitations of traditional solid microspheres—such as small size and limited field of view—when imaging large sample areas. However, most microspheres exhibit relatively low biocompatibility. Therefore, using a single biological element as a photonic component with distinct characteristics has emerged as an interesting new paradigm in biophotonics research. Notably, Chen et al.^42^ found that lipid droplets in mature adipocytes can serve as fully biocompatible microlenses to enhance microscopic imaging and detect both intracellular and extracellular signals. Moreover, they used OTs to manipulate lipid droplets for target localization and real-time imaging within cells. Zhai et al.^43^ proposed a novel microdroplet-assisted imaging technology based on OTs and microfluidic chips. By leveraging the light-generation characteristics of OTs, their system can produce droplets of various sizes, achieve different fields of view, and perform magnified imaging. Compared with solid microspheres, droplets still lack the same super-resolution imaging capability, but they offer controllable generation and a larger imaging field of view, potentially providing new directions for the development of microsphere-assisted imaging. Furthermore, by adjusting the viscosity of the droplet or the surrounding solution, the magnification of droplet-based imaging can be further enhanced. The droplet-generation scheme based on OTs is illustrated in Figure 5A. The relative position between the sample chamber and the objective lens can be adjusted at the micrometer scale using a displacement stage.

**Figure 5:**
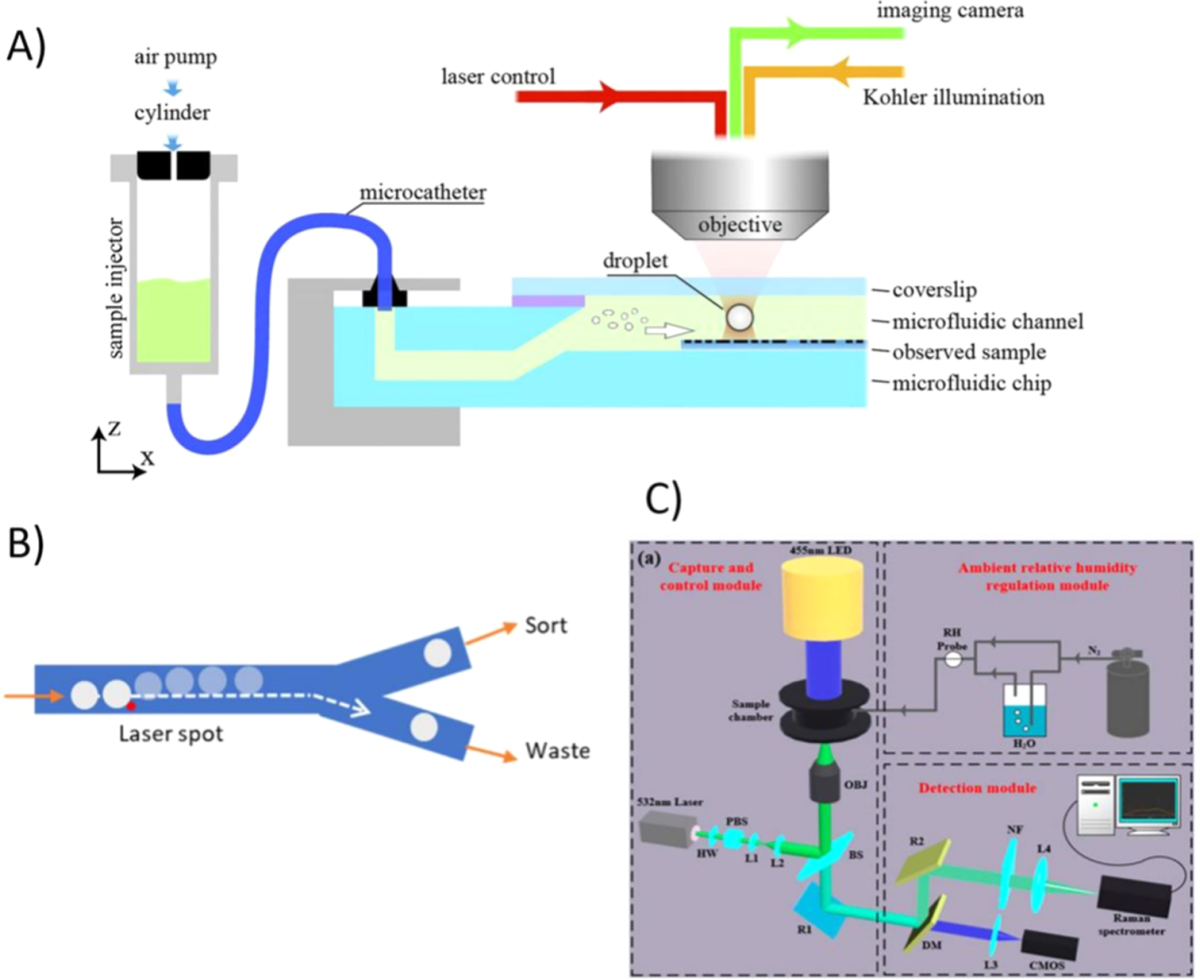
(A) OT-based microdroplet generation scheme (Reproduced from ref 43 Available under a CC-BY 4.0 license. Copyright 2023 Zhai et al.). (B) A laser beam focused spot can generate a localized thermocapillary effect, so that force can be generated at the oil–aqueous interface, to deter the trajectory of droplets (Reproduced from ref 45 Available under a CC-BY 4.0 license. Copyright 2023 Huang et al.). (C) Schematic of the microdroplet formation system based on OTs. HW: half-wave plate; PBS: polarizing beam splitter; L: lens; BS: beam splitter; OBJ: objective; R: reflector; DM: dichroic mirror; NF: notch filter; LED: light-emitting diode; CMOS: complementary metal-oxide semiconductor; RH: relative humidity; N2: nitrogen (Reproduced from ref 48 Available under a CC-BY 4.0 license. Copyright 2022 Li et al.).

Meanwhile, OTs can be integrated with fluorescence imaging, Raman spectroscopy, and microfluidics to enable the sorting and detection of droplets. A major goal of many optical manipulation techniques is to achieve practical functionalities, such as label-free sorting of biological cells—a field with a long history and promising prospects, as noted in a recent Outlook article.^44^ Like cells, droplets are soft matter. A single droplet can act as a microreactor, thereby allowing droplet sorting. For example, Huang et al.^45^ highlighted in their review that OTs combined with microfluidics can alter droplet trajectories through laser-induced thermal capillary effects or accomplish droplet sorting and splitting via resistive heating­(Figure 5B). Wang et al.^46^ developed a microfluidic sorting platform that integrates OTs with real-time fluorescence imaging, achieving a sorting purity of 94.4% for 10 μm fluorescent particles. This provides a new approach for the research and development of high-resolution microfluidic systems. Beyond droplet sorting, Tong and Ye^47^ studied individual biological particles in both liquid and gas phases using an integrated system of OTs and Raman spectroscopy. Li et al.^48^ utilized OTs to capture microdroplets at the center of the optical trap. By adjusting the experimental parameters and fitting Mie scattering to the spectral peak positions during the controlled growth process, they analyzed the Raman spectra and achieved both controlled growth and real-time characterization of the microdroplets­(Figure 5C). Overall, these technologies have advanced droplet manipulation from simple transport to intelligent platforms capable of in situ detection and feedback-controlled operations.

Additionally, OTs have evolved from simple trapping to enabling precise rotational control of objects. The torque OTs system introduced by the Bustamante team^49^ is a prime example, capable of simultaneously measuring torque, angle, force, and displacement. Wu et al.^50^ achieved full three-dimensional control of optical torque on trapped particles by manipulating the spatiotemporal distribution of vector spin angular momentum (SAM), as illustrated in the Figure 6A.

**Figure 6:**
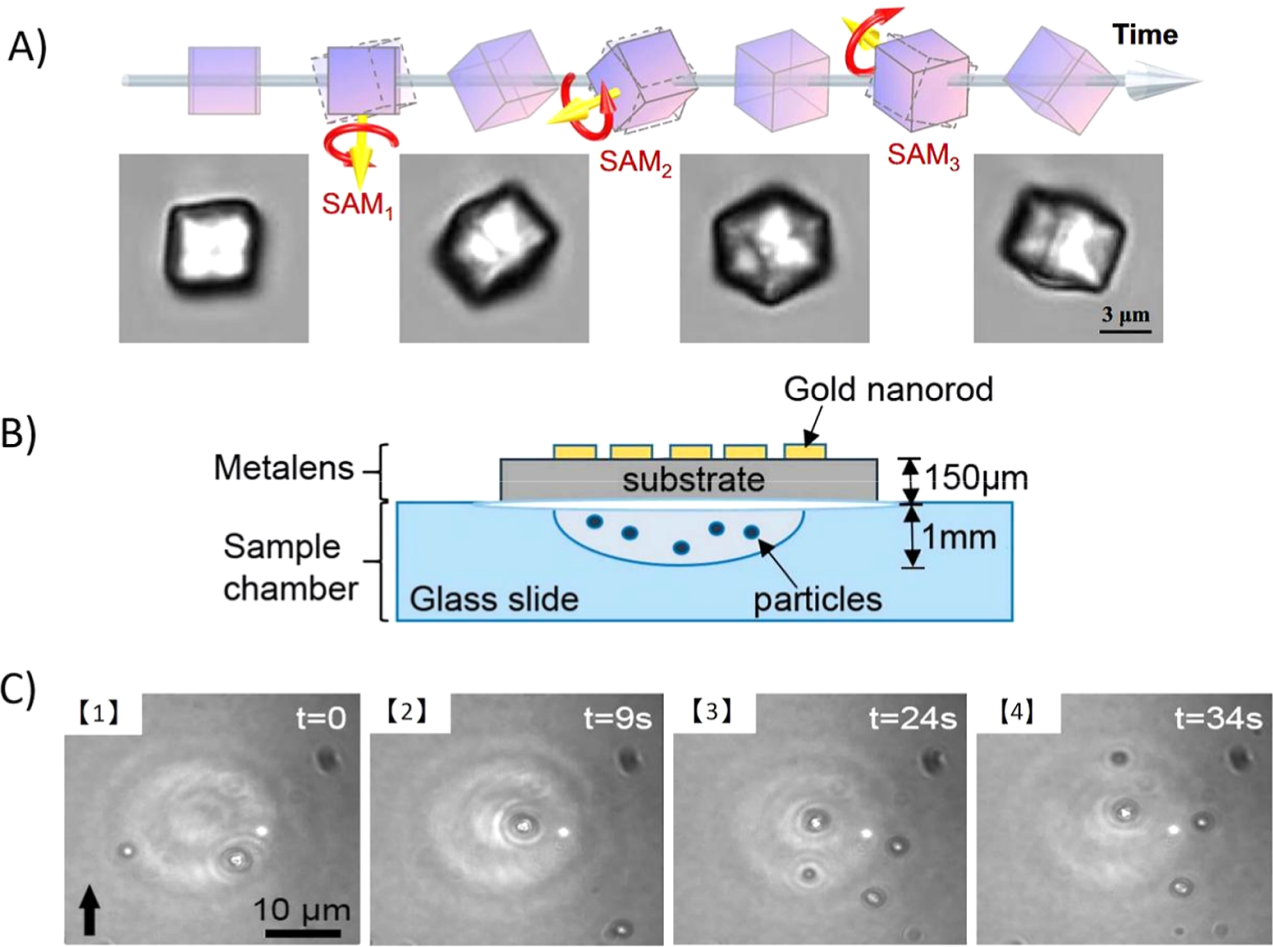
(A) Dynamic 3D optical spanner via time-varying vectorial transfer of the SAM to microparticles in an optical trap. Experimental images of a spinning cubic calcite particle demonstrate controlled rotations around an arbitrary axis in 3D space (Reproduced from ref 50 Available under a CC BY-NC-ND 4.0 license. Copyright 2025 Wu et al.). (B) Schematic sketch of the chambered glass slide, which was attached on top with metalens as a sample container (Reproduced from ref 51 Available under a CC-BY 4.0 license. Copyright 2019 Suwannasopon et al.). (C) A video sequence showing­(1,2) and NLC droplet upward due to heat convection and was trapped by metalens based laser tweezers­(3,4) The trapped droplet remained in the laser focus while others in the background kept flowing upward (Reproduced from ref 51 Available under a CC-BY 4.0 license. Copyright 2019 by Suwannasopon et al.).

Beyond the direct transfer of optical angular momentum, rotational motion can also be induced through specialized light–matter interactions. A notable example is the rotation of nematic liquid crystal (NLC) droplets. Leveraging the unique properties of NLCs and advanced optical techniques, such systems hold revolutionary potential for microfluidics, optics, and biological research. Suwannasopon et al.^51^ demonstrated NLC droplet rotation by integrating a metalens with OTs. Ultrathin metalenses, promising for future lab-on-a-chip systems, combine effectively with liquid crystals to form ideal microscopic optical motors for motion and flow control in various microsystems. Figure 6B presents the sample chamber with the integrated metalens, Figure 6C shows trapped NLC droplet by metalens based laser tweezers due to heat convection.

Further advancing this approach, Saito and Kimura.^52^ developed an optically driven NLC droplet rotator by combining elliptically polarized laser light with OTs. Their study analyzed the underlying rotation mechanism based on the internal arrangement of liquid crystal molecules. For NLC droplets, rotation is primarily driven by waveplate effects and light scattering, while for cholesteric LC (ChLC) droplets, it is explained by a combination of waveplate effects and Bragg reflection.

One potential future direction for enhancing manipulation of microdroplets via OTs is use of open microfluidic channel systems^53^ such as by Khor et al., which allows direct manipulation of droplets with PTFE - coated tweezers. The coating prevents strong adhesion of aqueous droplets to the tweezers, thus also facilitating droplet release. The PTFE-coated tweezers enables both lateral and vertical (picking up droplets) droplet transport in the microfluidic system. In the future, this approach could be used to sort droplets based on a reaction outcome or transfer droplets to a different part of a chip for further usage, all of which could be beneficial for many fields such as chemical analysis, drug delivery, and biological research.

Another potential lies in employing acoustic instead of OTs. In their study, Lin et al. successfully used single-beam (focused beam/vortex beam) acoustic tweezers^54^ as a selective sparse sampling method for W/O droplets. They generated the droplets with fluorescein dye (for later visualization and analysis), by using of a microfluidic device with flow-focusing junction and droplet sizes ranged from 20 to 150 μm. Droplets smaller than half a wavelength could be trapped by acoustic vortices, while larger water droplets could be trapped via focused acoustic beams. This enabled targeting and extracting selected droplet microreactors based on their size in the microfluidic system and analyzing their content. There is great future potential of using acoustic tweezers for droplet manipulation in combination with microfluidics in many different fields. This setup is especially useful for enhancing high-throughput drug screening assays, fluorescent labeling is not required for sorting and droplet handling is gentle.

### Stability of Emulsions

The stability of emulsions is essential for many industrial productions. The understanding of emulsion stabilization mechanism relies on the understanding of interaction forces between single droplet coated stabilizers.

OTs can be used to understand fundamental colloidal properties and their role in emulsion stabilization. The electrostatic interactions between highly charged particles dispersed in electrolytes have been obtained. Among the others, Crocker and Grier^55,56^ used OTs to measure the pair potential. A variety of measurements revealed purely repulsive interactions, which were quantitatively consistent with predictions of the DLVO theory. Elmahdy et al.^57^ studied the forces within single pairs of charged colloids in aqueous solutions of ionic liquids using OTs. The force curves were described by a size-corrected screened Coulomb interaction method. They obtained effective surface charge density from force curves, which altered with concentration and pH.

Additionally, OTs can be used to measure the elastic properties of polymers, Gutsche et al.^58^ summarized a review about microrheology on (polymer-grafted)­colloids using OTs. They presented several novel microrheological and microfluidic experiments, discussed force measurements and nonlinear responses within single pairs of DNA-grafted colloids, and even analyzed the drag force on colloids pulled through a polymer solution. Mahdy et al.^57^ and Dominguez-Espinosa et al.^59^ used OTs to measure a steric interaction of less than 20 pN between polymer brushes and clarified the entropic (osmotic) contribution of the counterions in the brush layers. Murakami et al.^60^ examined the long-range electrostatic interaction between polyelectrolyte brush surfaces directly using OTs.

Depletion interactions also play a significant role due to their influence on the behavior and stability of colloidal systems. Therefore, it was vital to quantitatively measure depletion interaction and predict the stability of colloids in advance. Liu et al.^61^ quantitatively measured the interaction forces (including depletion) between two silica particles induced by an ionic surfactant sodium dodecyl benzenesulfonate (SDBS) using OTs. Figure 7A shows force vs separation for a couple of silica particles during both the approach and retraction processes phases with SDBS micelles. Force hysteresis occurred in the retraction portion of the curve when the concentration of SDBS is larger than critical micelle concentration (CMC). The interaction forces between the same couple of silica particles with and without SDBS were measured in situ. By subtracting the force curves between the same couple of silica particles in the absence of SDBS from the force curves in the presence of SDBS, the pure depletion force can be quantitatively calculated. Figure 7B shows the calculated depletion forces.

**Figure 7:**
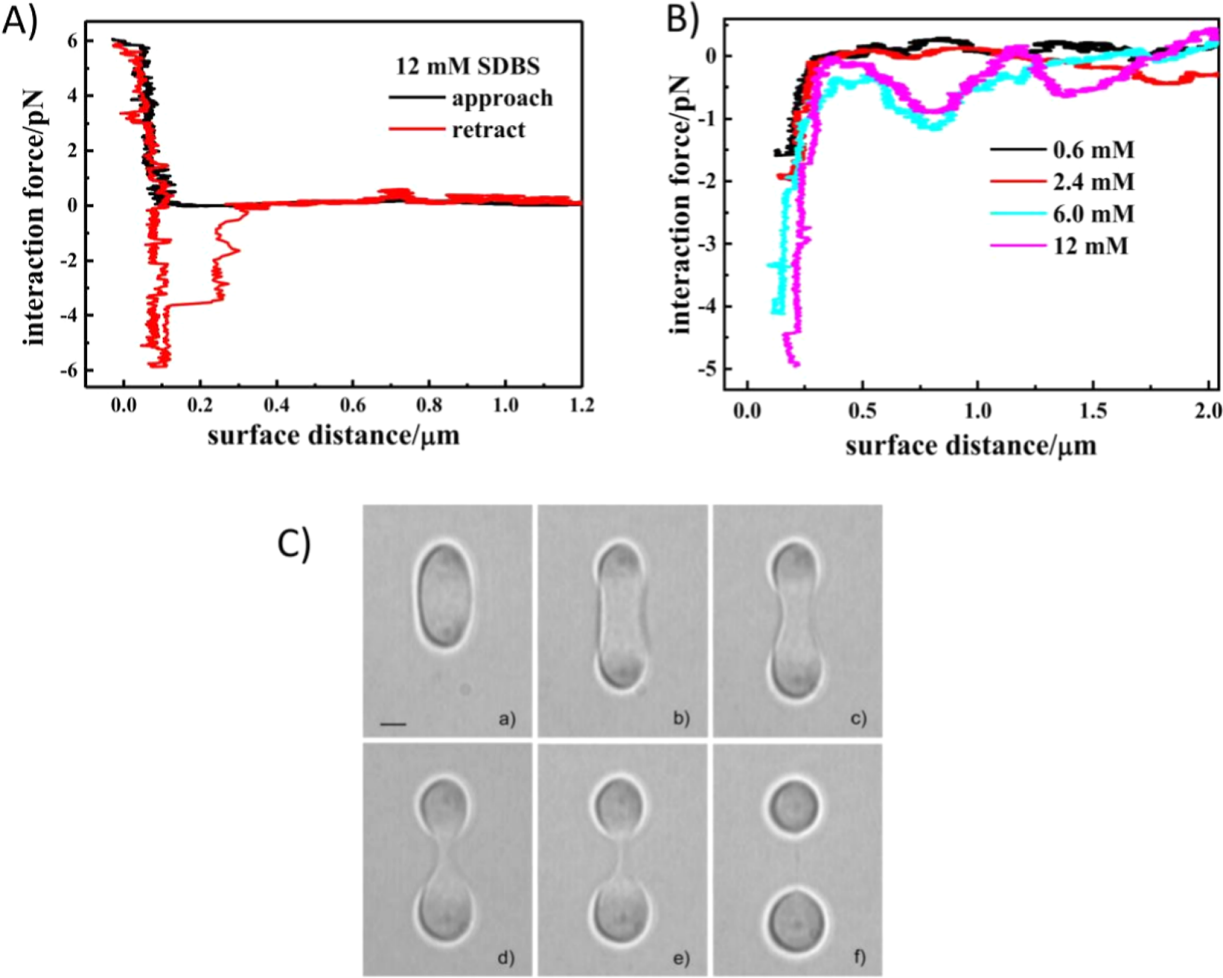
(A) Force vs separation for one single pair of silica particles SDBS at 12 mM, the approach (black line) and retraction (red line) (Reproduced from ref 61 Copyright [2019] American Chemical Society). (B) Calculation depletion forces between silica particles with the presence of different concentrations of SDBS (Reproduced from ref 61). Copyright [2019] American Chemical Society). (C) WitAmerican Chemical Societh very low tensions the droplets form dumbbell shapes on extension and can be separated and rejoined (Reproduced with permission from ref 14 Copyright 2006 Royal Society of Chemistry).

Several theoretical models have been developed to analyze interactions between colloids, which can serve as a basis for studying emulsion interactions and stability. Ward et al.^14^ reported an optical deformation technique for micron-sized O/W emulsions with ultralow interfacial tensions, achieved by the manipulation of multiple optical trapping sites within the droplets. They used this technique to characterize interfacial properties in the emulsion. The results were in good agreement with those obtained by Aveyard et al.^62^ and Mitani and Sakai.^63^ using the spinning drop interfacial tensiometry. Figure 7C shows multiple deformed droplets.

In 2014, Nilsen-Nygaard et al.^64^ qualitatively measured interactions between emulsion droplets for the first time. They compared force curves between emulsion droplets stabilized by micro- and macromolecular emulsifiers and explored the effects on depletion interaction. They observed the phenomenon that the biopolymer layer of sugar beet pectin (SBP) covering the emulsions surface reorganized during compression. Figure 8A shows the force versus time curve between two emulsion droplets stabilized by highly methylated SBP. There is a steady increase in repulsive force as the droplets approach due to the initial overlap of electric bilayer. At a certain point in the approach segment of the curve, the force suddenly drops to a lower level. After retraction, the force re-established at the same maximum level. The force reduction is reversible upon retraction, and coalescence of the droplets does not occur, indicating the rearrangement of the polymer layer. The force curves from OTs display the dynamics of macromolecular emulsifier layer. Additionally, attractive van der Waals forces can be measured during nondeforming polystyrene beads measurements, as shown in Figure 8B. Although they did not quantitatively analyze the force curve, these results are promising and imply that OTs can be a useful tool for researchers in the exploration of emulsion droplet interactions and stability.

**Figure 8:**
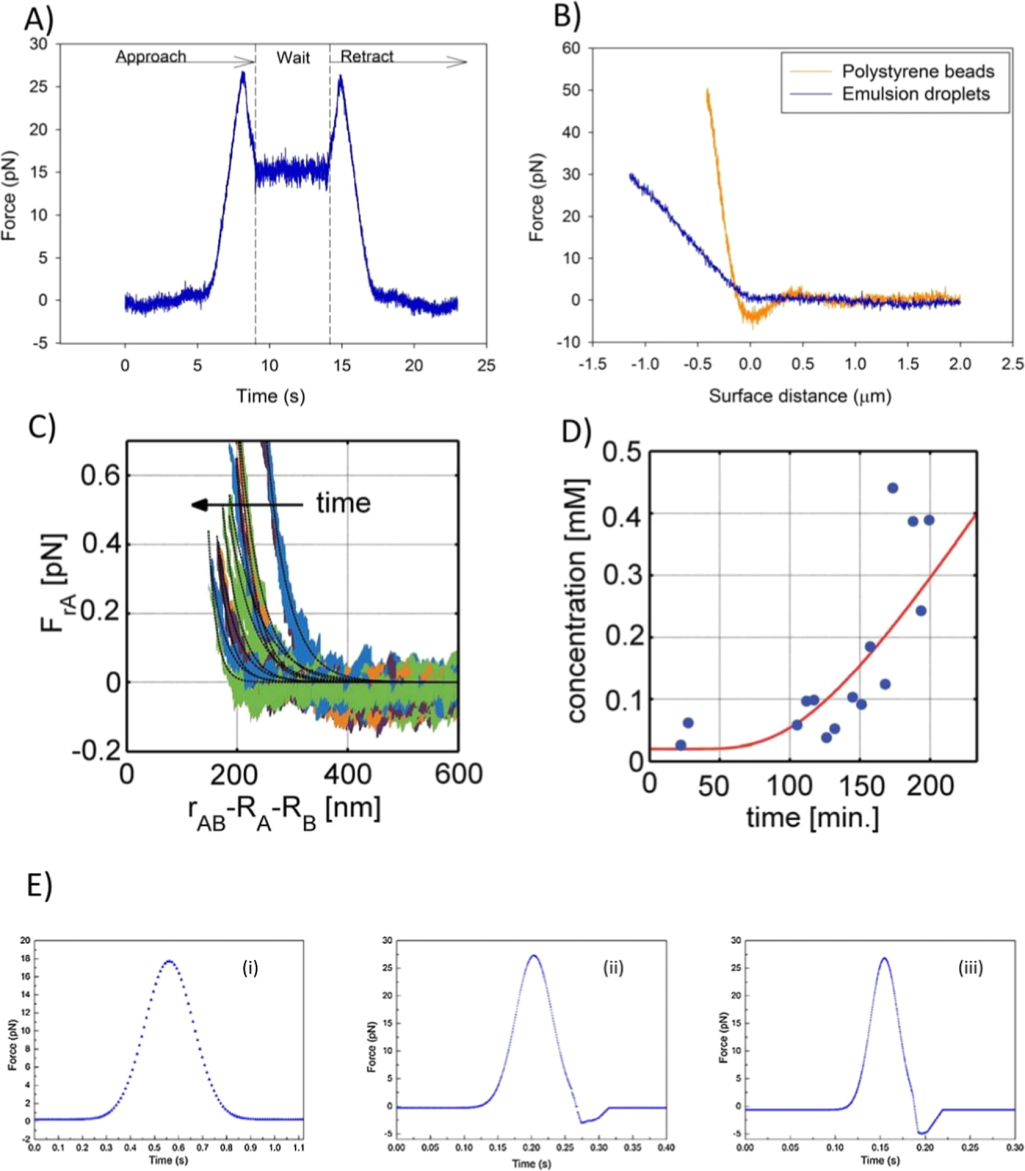
(A) Approach and retract curve for SBP stabilized droplets in MQ water (Reproduced with permission from ref 64 Copyright 2014 Royal Society of Chemistry). (B)­Approach curves for compression of a pair of rapid beads (2.7 μm) and a pair of emulsion droplets (2.5 μm) in MQ water (Reproduced with permission from ref 64 Copyright 2014 Royal Society of Chemistry). (C) Force–separation curves of emulsion droplets as a function of time as the local salt concentration was increasing owing to the diffusion of ions from an interface with a 5 mM salt solution (Reproduced with permission from ref 12 Copyright 2016 Royal Society of Chemistry). (D) The salt concentrations extracted from the time-resolved force data as a function of time (Reproduced with permission from ref 12 Copyright 2016 Royal Society of Chemistry). (E) Dynamic interaction forces between tetradecane droplets in three different approaching velocities (Reproduced with permission from ref 65 Copyright 2018 Elsevier).

Salt is known to affect the electrostatic force between two colloidal particles. An increase in salt concentration will reduce the Debye Length, leading to shorter distances of repulsion, which usually causes instability of the colloidal solution. Griffiths et al.^12^ proposed a novel method to measure local salt concentration using OTs based on this principle. A single couple of particles or emulsion droplets were kept in a microfluidic channel close to an interface formed between Milli-Q water and a 5 mM NaCl solution. As ions gradually diffused away from the interface, the salt concentration gradually altered, causing the force–separation curve to shift over time, as shown in Figure 8C. The Debye length can be fitted from the force–separation curves, and the local salt concentration can be obtained using eq 17, as demonstrated in Figure 8D, which was consistent with a relevant diffusion equation.

17 $$\kappa^{-1}=0.304/{c_{bulk}}^{0.5}$$

In recent years, Chen et al. have conducted many research studies, focusing on the measurements and analysis of interactions between micron-sized droplets.^65–67^ In 2018,^65^ they measured the interaction forces between tetradecane droplets in different concentrations of Sodium dodecyl sulfate (SDS) and NaCl solutions. They found that the droplets coated SDS are negatively charged and the EDL force gradually decreases with the increase of NaCl concentration. Additionally, they observed that absorption amount of the surfactant at the oil–water interface increases with the increase of SDS concentration. In these experiments it was possible to observe the “hydrodynamic suction effect” when the approaching velocity is increased, as shown in Figure 8E. The deformation ratio of emulsion droplets with a diameter of 5 μm is calculated, which means that almost no deformation occurs in the measurements.

In 2019,^67^ the same authors found that the tetradecane coated with non ionic surfactant FS-30 is negatively charged even though no ionic species were present in the system. Additionally, the screening effect of Ca^2+^, and Ba^2+^ on the EDL between droplets is stronger than that of Na^+^, which can be explained by the DLVO. In 2020,^68^ they established the quantitative relationship between the force and the separation distance between droplets using OTs and compared the measurement differences between AFM and OTs. Additionally, a numerical model has been demonstrated to calculate the repulsive pressure from the force curve. The repulsive pressure has the same expression for different sizes of droplets, as it is only a function of the interface separation distance. This model enabled to quantify the measured force between two micron-sized oil droplets coated with polymers and to better understand the interaction mechanism.

A great deal of work has reported how micelles can induce depletion attraction between two colloids. However, the effect of different micelles on the depletion attraction between two emulsion droplets has been rarely reported. Liu et al.^69^ explored the effect of different micelles on the depletion between two soft surfaces using OTs in 2022. Attractive forces between two like-charged emulsions could be measured. However, for nonionic surfactants, the attractive force between O/W emulsion droplets could not be measured even at the CMC of surfactant concentration. The results can explain how surfactant micelles would cause flocculation of emulsions by measuring depletion attraction force between a couple of emulsion droplets in situ. Moreover, it can be used to prepare stable emulsions by adjusting the types and concentration of surfactants.

Another factor affecting emulsion stability is pH, which especially plays a vital role in emulsions for nutrient and drug delivery applications, such as with oleic acid. At pH below 6.5, oleic acid forms oil-like structures, while at higher pH values, it forms O/W emulsions with complex internal nanostructures. Oleic acid is mostly known for usage in common oils but also shows potential for usage in drug delivery systems due to its response to pH.^70^ Manca et al. combined a custom-built platform with OTs, polarized optical video microscopy, microfluidics, and small-angle X-ray scattering to investigate the specific mechanisms behind this pH response and the structural changes and interactions among oleic acid molecules. Results showed that depending on the pH, oleic acid molecules go through different phases such as multilamellar vesicles, bicontinuous cubic structures, and hexagonal structures, while also exhibiting self-rotation due to changes in surface tension. For investigation of the interactions between oleic acid particles, the same authors also used double trap OTs. This highlighted that the force of roughly 100 nN applied by the OTs was not strong enough to cause the particles to coalesce, or merge together even at pH as low as 4.0. The same customized setup was also further used by the authors to gain insight into pH-triggered colloidal transformations that play a vital role in e.g. human lipid digestion and drug delivery systems.^71^ The authors investigated triolein digestion at single particle level by positioning a digesting triolein droplet inside a microfluidic chip via holographic OTs. Via the chip, pH and pancreatic lipase (an enzyme involved in fat digestion) levels were controlled, while microscopy and small-angle X-ray scattering were used to observe changes in morphology and structure of triolein.

It is worth mentioning the janus particles, which offer unique advantages in stabilizing emulsions due to their amphiphilic nature and precisely tunable surface properties. Unlike molecular surfactants, Janus particles can be designed with controlled hydrophilic/hydrophobic domain ratios and surface chemistries, enabling them to adsorb strongly at liquid–liquid interfaces and resist coalescence. Recent studies have further demonstrated that optical fields can enhance the functionality of Janus particles in emulsion systems, providing new avenues for real-time manipulation and stabilization.

For instance, the manipulation of Janus particles using evanescent fields near optical nanofibers allows precise spatial control, which can be utilized to direct these particles to emulsion interfaces for targeted stabilization.^72^ Similarly, plasmon-enhanced optical trapping techniques enable low-power, high-precision manipulation of Janus particles in aqueous or oily phases, facilitating their assembly at droplet interfaces with controlled orientation.^73^ The ability to optically position and reorient Janus particles in real time offers a dynamic method to modulate emulsion stability and droplet morphology.

The stabilization performance of Janus particles is governed by their structural parameters, such as the Janus structure parameter (JSP), which describes the relative size of the hydrophilic domain. Studies show that when the hydrophilic–hydrophobic contrast is significant, particles with a JSP below 0.48 tend to stabilize W/O emulsions, whereas those with a higher JSP favor O/W systems.^74^ This tunability, combined with the possibility of optical field-assisted localization, enables the design of responsive emulsions whose stability can be adjusted on demand.

Moreover, millimeter-scale PE stabilized by Janus particles exhibit enhanced stability against coalescence, especially when particles possess balanced domain ratios and optimal sizes.^75^ Optical trapping and patterning techniques can further assist in arranging such particles at interfaces, promoting the formation of nonspherical, jammed emulsion droplets with improved mechanical integrity.

OTs provide a powerful platform to investigate droplet stability by probing nanoscale interfacial phenomena, such as surfactant dynamics, thin-film drainage, and capillary forces between neighboring droplets, revealing how microscopic interactions dictate coalescence or stabilization.^76,77^ By correlating these real-time, high-resolution measurements with macroscopic emulsion properties^78^ (e.g., shelf life, rheology), researchers can establish design rules for optimizing stabilizers (e.g., nanoparticles, polymers) or tuning interfacial elasticity, enabling rational emulsion engineering for applications in drug delivery, food science, or soft materials, where precise control over droplet stability is critical.

### Aggregation and Coalescence of Emulsion Droplets

The mechanisms of emulsion aggregation and coalescence are particularly important in the food industry. In food, emulsions can produce different effects.^79–81^ On the one hand, for food products such as sauces and milk products, aggregation and coalescence should be avoided to extend shelf life and ensure quality and consumer satisfaction. On the other hand, for products such as ice cream, whipped cream or butter, partial coalescence is required to ensure correct structure formation for the desired sensory properties in the sample preparation protocol.^82^ While partial coalescence in food emulsions has been widely studied, the mechanism of stabilization of different partially coalesced states has not been fully understood.

Recently, Mitsunobu et al.^83^ examined the coalescence of oil droplets stabilized by a surfactant or a hydrophilic polymer using OTs. They observed that droplets could not coalesce at room temperature in spite of the type of emulsifier. In contrast, the coalescence of droplets stabilized by the neutral hydrophilic polymer polyethylene glycol (PEG) was achieved at a temperature higher than 30 °C. However, the droplets with ionic surfactants cetyltrimethylammonium bromide (CTAB) or SDS did not coalesce even at high temperature due to their electrostatic repulsion.

The solid content of viscoelastic emulsion droplets can influence their tendency to aggregate and their following coalescence behavior. The balance between the drive to reduce surface tension and the straining of an internal viscoelastic network can create a large number of stable partially coalesced states.^79^ R Otazo et al.^13^ studied the aggregation and subsequent partial coalescence of microsized anhydrous milk fat (AMF) droplets by combining OT and a temperature-cycling regime. AMF was chosen to prepare droplets to ensure the presence of crystals in the emulsion. They utilized OTs to make two partly crystalline droplets approach until the distance between them was smaller than the size of the protruding part of the crystal. They used a temperature-cycling regime to adjust the amount of fat crystal in the droplets, which allowed two approaching droplets to gradually merge and take on a spherical shape driven by the Laplace pressure. The use of OTs allows for real-time observation of the aggregation and coalescence processes of partially crystalline emulsion droplets at the microscale. This provides a detailed understanding of the dynamic interactions between droplets and quantitative data on the forces and temperature that lead to aggregation and coalescence. The experimental scheme is shown in Figure 9A. Figure 9B shows arrested coalescence at different temperatures.

**Figure 9:**
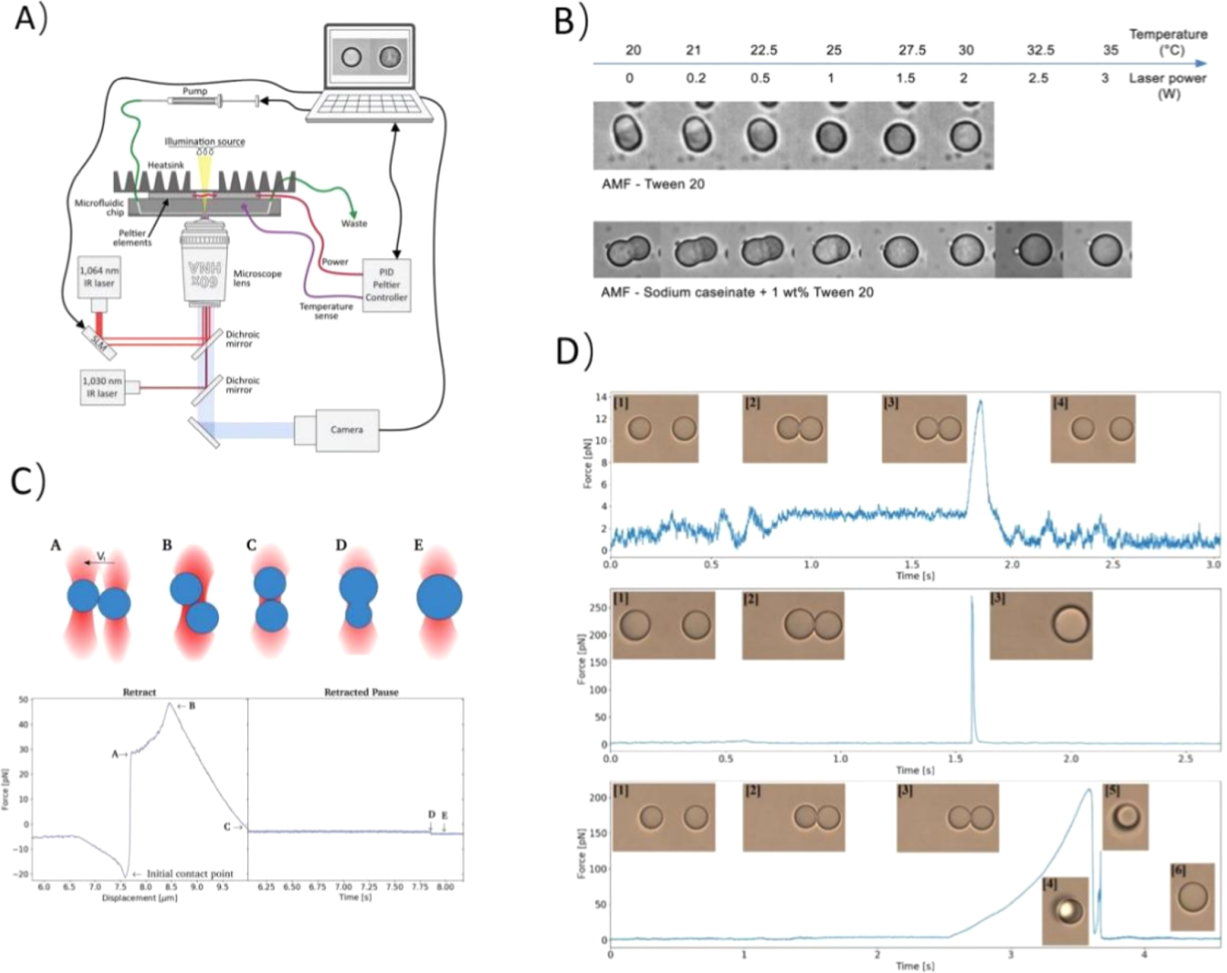
Illustrates (A) Peltier PID controller module fitted to an optical microscope with camera and laser tweezers (Reproduced with permission from ref 13 Copyright 2019 Royal Society of Chemistry). (B) Arrested coalescence at different temperatures by heating droplets with the HPL at different output powers (Reproduced with permission from ref 13 Copyright 2019 Royal Society of Chemistry). (C) Trap force versus time curves showing droplet behavior during retract–extend cycles obtained using OTs (Reproduced from ref 85 Available under a CC BY-NC 3.0 license. Copyright 2021 Aarøen et al.). (D) Top: insufficient contact between the droplets. Middle: excessive contact between the droplets. Bottom: sufficient contact between the droplets (Reproduced from ref 85 Available under a CC BY-NC 3.0 license. Copyright 2021 Aarøen et al.).

Droplet coalescence is also affected by concentrations of specific chemical solutions. This was exemplified by Wen et al., who, via their scanning OTs system, controlled coalescence and splitting of microreactors in femtoliter/picolitre droplets.^84^ Increasing ion concentration or exciting fluorescence caused oil droplets to coalesce, either due to the attraction of oppositely charged ions on the droplet surface or weakening of order of the oil molecule arrangement. By addition of an emulsifier and fluorophores into their liquid medium, Wen et al. could also split and stretch oil droplets via excitation of the fluorophores and OT forces.

Aarøen et al.^85^ investigates how approach velocity affects the likelihood and mechanism of coalescence, revealing the conditions under which droplets are more likely to merge. The depletion force between pairs of droplets was measured based on retract-extend measurements using OTs, which was used to avoid insufficient or excessive contact. Figure 9C shows Trap force versus time curves showing droplet behavior during retract−extend cycles obtained using OTs. Figure 9D shows the relationship between force and time during the droplet retract–extend cycles obtained by OTs, top picture shows insufficient contact between the droplets, where depletion force will not be observed during two droplets approaching. Middle picture shows an excessive contact, causing coalescence of two droplets during the pause^2^ (in Figure 9D insets), bottom picture shows sufficient contact, where the depletion force was high enough to rearrange two droplets in one trap, and coalescence occurs during^6^ (in Figure 9D insets). The coalescence time was defined as the time period from the first encounter between the two droplets until their rupture, as shown in Figure 9D. This is crucial for controlling emulsion properties, especially in processes where maintaining or breaking emulsions is necessary. Understanding the transient behavior of droplets as they approach each other at different velocities provides deeper insights into the stability and dynamics of emulsions over time. Aarøen et al.^86^ conducted a multidisciplinary study on thin film breakage in O/W emulsions, exploring the mechanisms and factors influencing the rupture of thin liquid films between droplets, which is crucial for understanding emulsion stability.

OTs enable precise manipulation and real-time observation of emulsion droplet aggregation and coalescence, offering insights into the role of interfacial forces, surfactant dynamics, and external stimuli (e.g., pH, temperature) in destabilization processes. These studies can guide the rational design of stable emulsions for pharmaceuticals (e.g., controlled drug release), food science (e.g., texture optimization), and cosmetics (e.g., shelf life extension), while also advancing fundamental understanding of colloidal interactions in soft matter systems.

### Switchable Behavior of Responsive Emulsions

In some applications, stable emulsions are only temporarily preferred in a certain stage and followed by a controlled demulsification process, which have attracted widespread research interest in various industrial fields including drug delivery, oil transport, and fossil fuel production.

In recent years, switchable or stimuli-responsive emulsions^87^ were demonstrated and reversible switch between “emulsification” and “demulsification” by external stimuli or triggers (such as pH,^88,89^ temperature,^90,91^ light irradiation,^92^ redox,^93,94^ magnetic field,^95^ CO_2_/N_2_,^96^ or multiple stimuli^97^) were reported. The core process of these systems is the switchable behavior between emulsification and demulsification, which is inseparable from the stability and instability of the emulsions. The quantitative measurements and analysis of interactions between a pair of switchable emulsion droplets are urgently desirable.

Switchable surface-active colloid particles are crucial for the preparation of switchable Pickering emulsions (PE).^97–100^ Researchers can explore particle dynamics, assembly processes, and phase transitions as a basis for extending OTs techniques to the study of emulsion droplets and other complex systems. In general, the initial colloidal particles are usually so hydrophilic and surface-inactive that they could not prepare stable PE. To solve these limitations, many previous studies have provided effective methods for partially hydrophobized colloidal particles by adsorbing switchable surfactants with opposite charges, enabling the preparation of switchable PE through certain triggers. Chen et al.^101^ developed a novel approach to measure the interaction forces between a couple of switchable surface-active colloid particles in situ using OTs. They prepared switchable surface-active silica particles by partially hydrophobizing commercially available inorganic silica particles in water using the common cationic surfactant CTAB. Furthermore, the surface-active form can be converted to the surface-inactive form at room temperature by using the conventional anionic surfactant SDS. Figure 10A shows a diagrammatic sketch of force measurement between switchable surface-active silica particles. Figure 10B showed interaction forces of switchable surface-active colloid particles.

**Figure 10:**
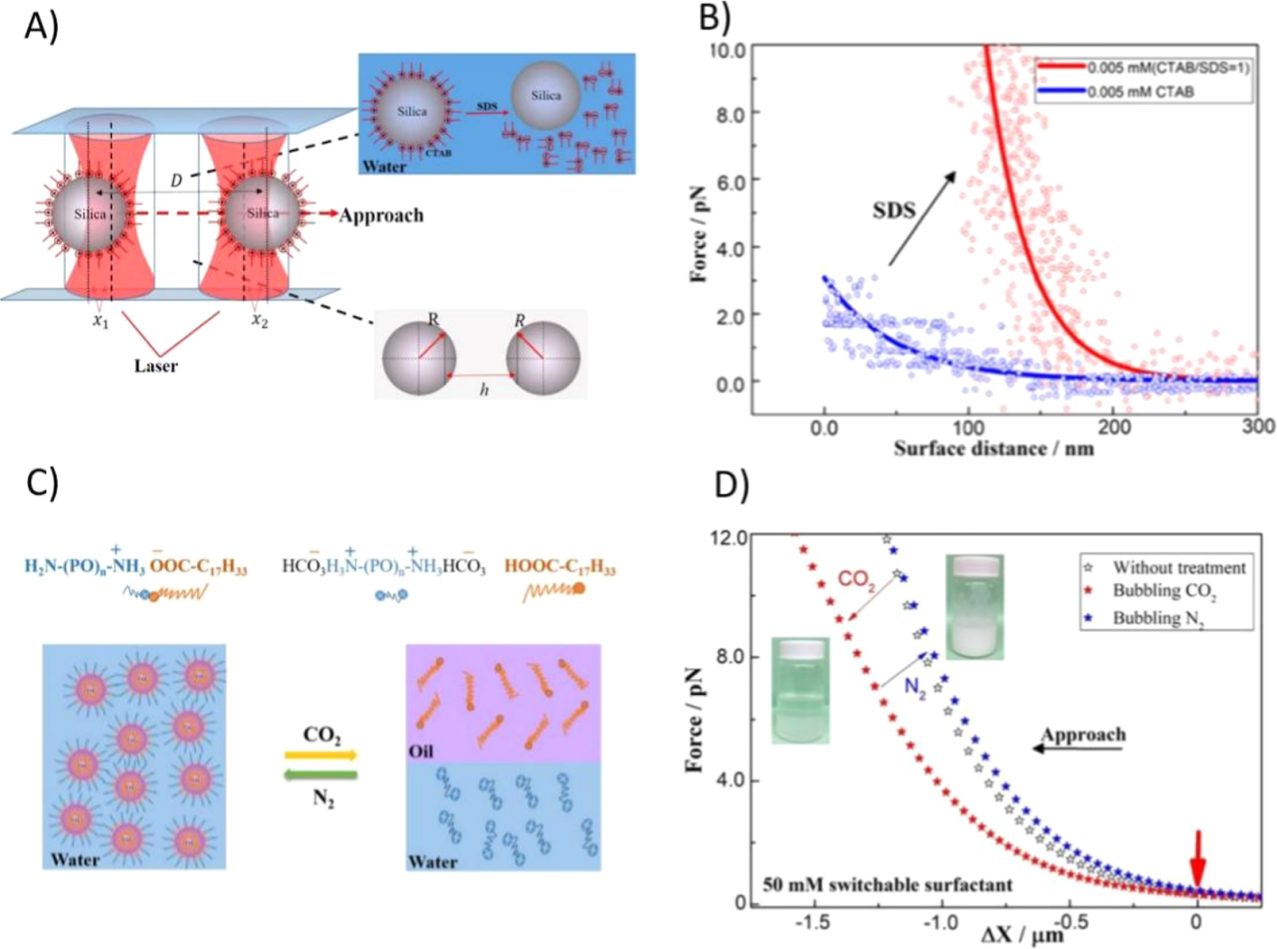
(A) Diagrammatic sketch of force measurement between switchable surface-active silica particles (Reproduced from ref 101 Copyright [2020] American Chemical Society). (B) Interaction forces between two 5.0 μm silica particles in 0.005 mM CTAB solution or with the addition of equimolar SDS molecules (Reproduced from ref 101 Copyright [2020] American Chemical Society). (C) Schematic diagram of the measurements of interaction force between a couple of individual switchable emulsion droplets and the process of stimulus responsivity of the switchable surfactant by CO_2_/N_2_ trigger (Reproduced from ref 103 Copyright [2020] American Chemical Society). (D) Force curve of 5.0 μm (diameter) tetradecane droplets between the processes of emulsification and demulsification upon bubbling CO_2_ or N_2_ alternatively (Reproduced from ref 103 Copyright [2020] American Chemical Society).

Bauer et al.^102^ combined the concepts of engineered emulsions with the advantages of the microfluidic methods. It is possible to generate monodisperse, functional O/W droplets stabilized by a pH-responsive copolymer surfactant in microfluidic devices. Aggregation and disaggregation driven by interdroplet hydrogen bonds formed macroscopic structures and dispersed structures, which were controlled by a simple pH trigger. PH-dependent interactions between individual droplets were quantitatively analyzed using OTs.

Chen et al.^103^ measured the interaction forces between the CO_2_-responsive switchable behaviors of demulsification and restabilization using OTs and revealed the switchable mechanism. They introduced CO_2_ and N_2_ into emulsion droplets and achieved detachment/self-assembly of the switchable surfactant, which caused the desorption and reabsorption of the switchable surfactant from the water–oil interface, leading to the weakening and re-enhancing of the EDL repulsive forces between emulsion droplets. Figure 10C shows schematic diagram of the measurements of interaction force between a couple of individual switchable emulsion droplets and the process of stimulus responsivity of the switchable surfactant by CO_2_/N_2_ trigger. Figure 10D showed the force curve of droplets between the processes of emulsification and demulsification. Recently, Cheng et al.^104^ explored the use of light-responsive materials in droplet manipulation for biochemical applications, highlighting how these materials can enable precise control over droplet behavior through light-induced changes, facilitating various biochemical processes and analyses.

OTs studies of switchable emulsions directly advance smart drug delivery by optimizing triggered release mechanisms^105^ (e.g., light-responsive droplet rupture), adaptive coatings by designing emulsions that reversibly alter wettability or self-heal under stimuli, and programmable soft robotics by controlling emulsion-based actuators for shape-morphing or locomotion.^106^ These applications exploit precise, on-demand emulsion destabilization or stabilization, enabled by correlating microscopic tweezer data with macroscopic responsive behavior.

### Instrumentation

Previously, the applicability of OTs in relation to emulsions was discussed. OTs can achieve a higher efficiency in certain applications than current widely used methods. For instance, in isolation and separate encapsulation of individual cells, OTs are superior to a statistical approach to encapsulation, and also to commonly used sorting strategies.^107,108^ However, their spread is limited, one possible reason for which may be the complexity of their instrumentation. Another possible limitation is affordability, as OTs require a combination of high-end instruments to set up.^23^ In this section, we overview the instrumentation framework of various OT setups, and evaluate their complexity and cost with a view toward the democratization of this technology.

To compare OT setups in terms of cost and complexity, we take essential functional elements, as well as the main cost-drivers (Table 2). These are (1) objective, (2) number of beam-forming and beam-steering optical components (light sources and detectors are not counted, but the objectives are), (3) light source used for entrapment, and (4) the detector used for imaging.^23^ We also compare the application and the object the laser beams entrap. While the number of beam-steering optical components may not be directly comparable due to the diverse applications, it can be indicative of a difference (if there is one) between setups declared as “low-cost” in the literature and setups that are not. Applications in Table 2 are categorized using the ontology introduced in the subsections of this section.

**Table 2:** Comparison of OT Instrumentation Setups for the Manipulation of Emulsions

aggregation and coalescence of emulsion droplets		emulsion stability studies			manipulation of emulsion droplets					application
9	6	8	6	7	10	8	/	9	9	no. of optical components
40*x* (NA 0.6)	90*x* (60*x* Nikon MRD07602, NA 1.2 + auxiliary 1.5*x* lens)	100*x* (Nikon, CFI Plan 100XC W water-immersion, NA 1.10)	60*x* (Nikon Eclipse TE2000-U, NA 1.2)	60*x* (Nikon water-immersion, NA 1.0)	60*x* (Nikon water-immersion, NA 1.0)	100*x* (Nikon CFI Plan 100 XC W)	63*x* (ZEISS, NA:1.2)	60*x* (Olympus 60*x*, NA 0.7)	40*x* (for imaging)	objective
532 nm @ 3 W (Beamtech Optronics Nd:YAG laser)	1064 nm @ 2 W (diode laser, movable trap) and 1030 nm @ 5 W (diode laser, fixed trap)	1064 nm laser (Nd:YAG crystal laser, IPG photonics, model YLR-10-LP)	1064 nm @ 2 W (diode laser, movable trap) and 1030 nm @ 5 W (diode laser, fixed trap)	1064 nm @ 5 W (part of aresis instruments Tweez 250si)	1064 nm CW (part of aresis instruments Tweez 250si)	1064 nm (IPG photonics YLR-10-LP)	1064 nm @ 5 W (IPG photonics YLR-5-LP)	532 nm @ 50 mW for imaging, 1064 nm laser @ 150 mW for sorting	1064 nm @ 300 mW (circularly polarized CW fiber laser)	light source
oil droplets	AMF droplets	oleic acid droplets	silica beads; soybean oil droplets	silica particles	mature adipose cells with lipid droplets	triolein droplet	microdroplet	*E. coli*, yeast cells	NLC droplets (5CB-CTAB)	tweezed object
								*E. coli*, yeast cells		
								(F. accuracy)		
camera (Watec WAT-221S CCD)	camera (16 bit CMOS, Andor NEO)	camera (CCD)	camera (16 bit CMOS, Andor NEO)	camera (CCD)	camera (CCD) and spectrometer	camera (Nikon DS-Qi_2_)	camera (Manta G-507 monochrome)	camera (CCD)	camera (CCD)	imaging detector
		teledyne FLIR LLC Flea3 FL3-U3-13E4M			(SHIS VNIR-520-20 S)					
^83^	^13^	^70^	^12^	^61^	^42^	^71^	^43^	^107^	^51^	refs

As indicated by Figure 4, open-source/low-cost OT systems have entered publishing very recently, and systems for manipulating emulsions/droplets occurred first in 2020. In terms of instrumentation, however, they are similar, and thus we will report on both, starting with systems dedicated to the manipulation of droplets/emulsions. Suwannasopon et al.^51^ demonstrated a setup for driving NLC droplets (Figure 6B). It used a 1064 nm fiber laser operated at 300 mW, which was circularly polarized by a Glan-Thompson polarizer and quarter waveplate (QWP). NLC droplets were held in a glass slide chamber filled with 4.5 μm polystyrene beads, in direct contact with a metalens. A CCD camera was used to image the movement of the beads through a 40× objective. Xu et al.^107^ presented the EasySort system (Figure 11) for OT-assisted pool-screening and single-cell isolation (OPSI), that is, capture of individual cells (1–40 μm) and selective encapsulation in nanoliter droplets. The authors claimed >99.7% sorting accuracy with a throughput of 10–20 cells/min. While the sorting throughput is significantly lower than more traditional label-free droplet-based cell sorting methods (∼40 droplets/second), the accuracy is considerably higher (∼90–95%).^109,110^ Zhai et al.^43^ performed super-resolution microscopy by suspending microdroplets above the object under observation using an OT (Figure 5A). Compared to its utility, the experimental setup was fairly simple. An ytterbium fiber laser (IPG Photonics YLR-5-LP) was used with a high NA lens (ZEISS 63*x*, NA:1.2). The experimental setup of Chen et al.^42^ consisted of an inverted fluorescence microscope (Nikon Eclipse Ti), and a scanning optical tweezing system (Aresis Tweez 250si) and was used to manipulate lipid droplets in mature adipose cells.

**Figure 11:**
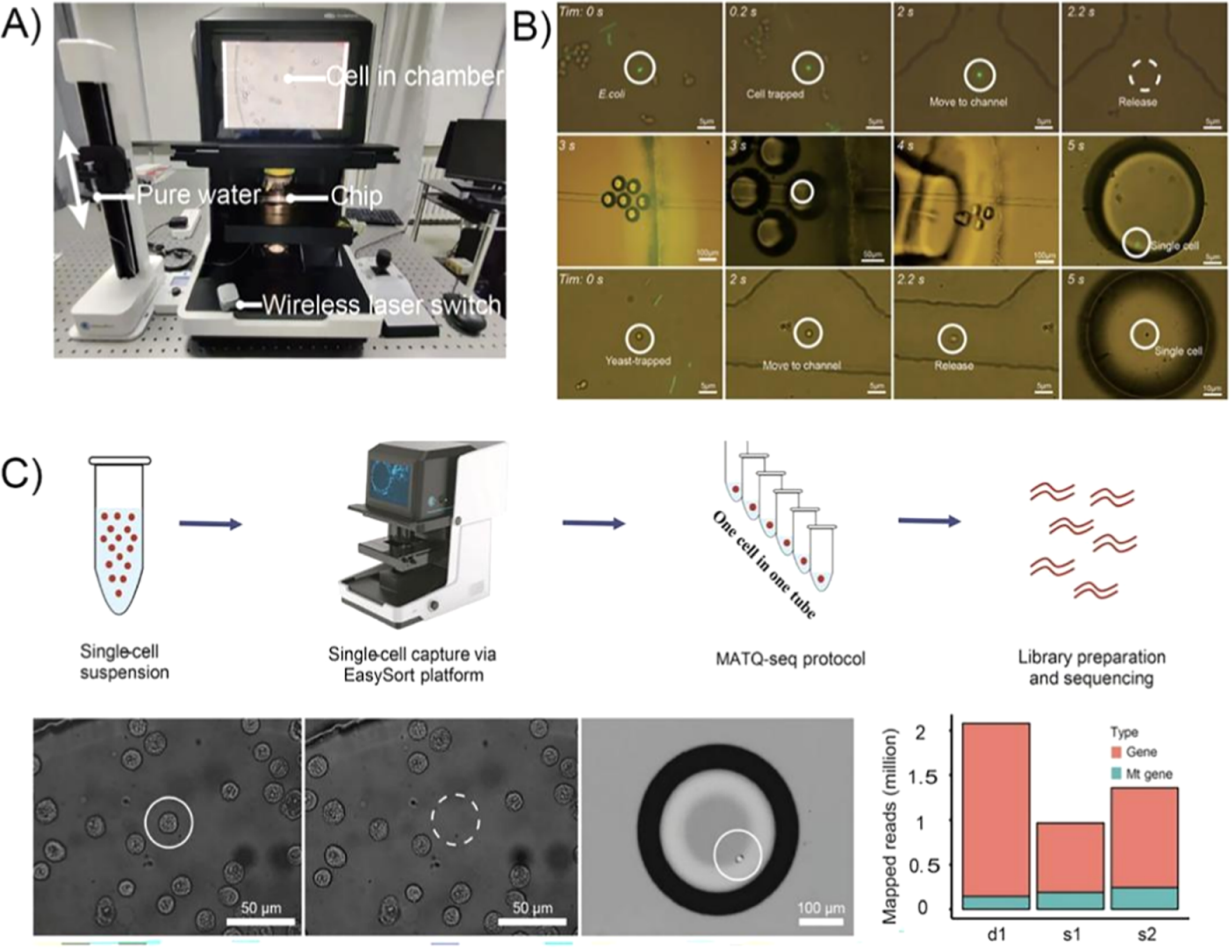
(A) EasySort platform ready for single-cell sorting.^107^ (B)­The single-cell sorting of artificial mock samples which contained *E. coli* and yeast.^107^ (C) A novel OPSI-seq workflow, which combines our OPSI platform with single-cell RNA-seq library preparation (Reproduced with permission from ref 107 Copyright 2022 Royal Society of Chemistry).

Interaction force measurements in emulsions is another unique application area of OTs. Several authors in this field used standard OTs instrument setups. For instance, Liu et al.^61^ measured interaction forces (Figure 7A) between microparticles in an emulsion using Aresis Tweez 250si system as Chen et al.^42^ with no apparent modifications to the optical path. Julie et al.^64^ used on a Nanotracker (JPK Instruments) mounted on an inverted light microscope (Zeiss Axio Observer A1), and Chen et al.^65^ used a Nanotracker 2 (JPK Instruments). Griffiths et al.^12^ built their setup on the basis of a HOT (Arryx Inc., Holographic OTs) system and used two diode laser beams, one movable (1064 nm, 2W) and one stationary (1030 nm, 5 W).

Aggregation/coalescence of emulsions is widely researched (Figure 4). R Otazo et al.^13^ also used the Arryx HOT system with the same dual laser beams, albeit with a different set of objectives with a higher magnification (60× Nikon MRD07602, NA 1.2 + auxiliary 1.5× lens resulting in a 90× total). Aarøen et al.^85^ used a Nanotracker 2 (JPK Instruments) mounted on a Zeiss Axio Observer Inverted optical microscope. The laser used was a TEM00 with a 3 W maximum power. Mitsunobu et al.^83^ also used an inverted microscope (Nikon TE2000-S) as the basis of their experimental setup, and outfitted it with a 532 nm, 3 W, Nd:YAG laser (Beamtech Optronics). The laser beam was split by two beam splitters, and the resultant beams were introduced to the microscope objective via dichroic mirrors.

Low-cost and open-source are new concepts in OTs, having started around 2019, with ∼5–10 new publications per year in the last 5 years (Figure 4). Some fields, such as droplet-based single-cell isolation, could significantly advance by means of emulsion OT technology. In these fields, more affordable instrumentation could provide a significant boost to application development.

At present, however, some limitations to this subfield exist: only particular components, such as software used for analysis are open-source, and are not self-developed, whereas self-developed hardware systems declared by the authors as “low-cost” typically have no cost calculation included for comparison. They also show little to no difference in terms of instrumentation, as compared to setups not reported as low-cost. Finally, publishing rate seems to decrease over time. There may be some technical challenges limiting increased growth in this research area.

### Optical Tweezers for Emulsions–Limitation in the Experimental Method

Although the use of micropipette and OT in single trap configurations was reported in the study of colloids,^57,59^ a dual-laser OTs is typically used to study couples of emulsion droplets. In a dual-laser configuration, one of the beads/emulsions (held in a steerable trap) is stepped toward the other beads/emulsions (held in a fixed trap). In order to exclude the effect of hydrodynamic force, the approaching velocity is usually adjusted below 1.0 μm/s,^65^ and when the two droplets get close to each other and start to interact, the force between them can be calculated.^68^

During sample preparation, emulsions tend to adhere to sample chamber making the trapping experiment difficult. To limit the adhesion between droplets and sample chamber, the surface treatments should be used. For instance, Nilsen-Nygaard et al.^64^ reported on the use of 1 mg/mL BSA solution to coat surface of the cover glass for 60 min, while Murakami et al.^60^ used a coating of (3-(2-aminoethyl)­aminopropyl)­trimethoxysilane to prevent the adsorption of particles onto the fluidic chamber surface. However, the identification of a more standard protocol to prevent adhesion would be preferable.

Another major challenge in using OTs for the study of emulsions is data reproducibility. In particular, the interaction of different droplets in different OT experiments can be significantly different due to differences in droplet size, and the adsorption of the emulsifier at the oil–water interface. The resolution of the light microscope does not always allow a precise determination of the contact point between droplet surfaces. In order to improve data reliability and reproducibility it is critical to obtain samples as chemically homogeneous as possible prior to emulsion experiments. For example, using a droplet generation device in microfluidic to obtain uniform droplets. Researchers did important efforts to obtain a measurement interval under a certain condition, such as the same type of emulsifiers, droplet size, approach velocity and solution properties, as mentioned by Nilsen-Nygaard et al.^64^ and Aarøen et al.^85^ Interestingly, OTs combined with microfluidic channels can achieve interaction measurement between the same pair of emulsion droplets in different environmental conditions in situ.^12,61,101^

Current studies focus on single-droplet manipulation (as typical in foundational OTs experiments). The stock solution is typically diluted according to experimental conditions, including salt concentration or pH.

The emulsion properties are time-dependent, therefore, in order to avoid coupling into more influencing factors, researchers will create relatively stable emulsions for a short period of time, and then actively change the environment of the emulsion to study the state of the emulsion under different conditions, including the movement of the emulsion, the interaction forces between droplets or coalescence, etc.

However, the current research methods still have certain limitations. Several potential statistical models are expected to be combined with OTs for studying droplet capture in complex emulsion, such as machine learning models,^111^ population balance models (PBMs)^112^ and Semiempirical models etc.^111^

OT-technology is not suitable for the trapping and analysis of droplets in W/O emulsion systems as the refractive index of the water phase is usually lower than oil phase. This limits the direct use of OT-s in droplet microfluidics where W/O droplets are increasingly being applied: e.g. in diagnostics,^6^ single cell analysis,^113^ screening for novel drugs^7^ and enzymes.^114^ However, one can easily envision OT-s being used together with droplet microfluidics for trapping and analyzing cells of interest for downstream encapsulation into W/O droplets and further analysis (e.g., genomics). Biological cells usually have higher refractive index than their surrounding water-based medium.^115^ OT-s have been shown already be effective in sorting cells of different sizes into water-droplets for further genomic analysis in low-throughput settings.^107^ The open challenge then remains to develop further the OT technology in combination with droplet microfluidics to enable such analysis in high-throughput. So far, various precise, flexible and high-throughput manipulation techniques have been developed. Optoelectronic tweezers (OET)^116^ is an advanced technique combining light stimuli with electric field together by utilizing the photoconductive effect of semiconductor materials, which can be used to manipulate water droplets in water. Additionally, the use of donut beams to trap low-index particles can be used to trap and manipulate oil in water droplet. Gahagan Swartzlander.^117^ already reported the low-index particle trapping in 1999. Garbin et al.^118^ reported on the use of donut beams to trap ultrasound contrast agent­(UCA) micro bubbles, thus it is possible to manipulate oil in water droplets through the use of donut beams.^119^

### Outlook

In conclusion, OTs have emerged as a potential tool for the study of emulsions thanks to its high spatial and temporal resolution and high sensitivity in measuring forces. Moreover, OTs enable the suspension of the emulsion in the specified position in the liquid and to control the environmental conditions of the emulsion.

To date, OT can be combined with emulsion droplet to form a special functional optical device, such as ideal optical motors and droplet-assisted imaging system. Additionally, the stability mechanism of emulsion stabilized by single emulsifier has been studied by measuring the force and displacement between two dispersed droplets by OT, even utilizing an instability mechanism to control aggregation and coalescence of emulsions. Moreover, switchable behavior of pH-responsive and CO_2_-responsive emulsions has been investigated.

The future of OTs in emulsion science lies in their evolution into a quantitative, multimodal platform uniquely positioned to answer unresolved core questions at the single-droplet level. Specifically, OTs enable the deterministic investigation of microscopic interfacial dynamics—such as the fundamental processes of droplet coalescence/fission and the real-time link between interfacial viscoelasticity and stability—which are inaccessible to bulk experiments. Furthermore, OTs are critical for probing nonequilibrium assembly pathways in active emulsions containing energy-consuming components. To overcome current limitations, emerging technologies are now directly linked to these scientific goals: machine learning decodes high-dimensional dynamics from droplet trajectories; advanced beam shaping manipulates multidroplet configurations and internal flows; and hyperspectral/thermal imaging correlates mechanical manipulation with in situ chemical and thermal changes. This integrated approach transforms OTs from a micromanipulation tool into a definitive platform for bridging interfacial mechanics, soft matter physics, and nonequilibrium chemistry in emulsion systems.
